# Structural characterization of piglet producing farms and their sow removal patterns in Finland

**DOI:** 10.1186/s40813-019-0119-8

**Published:** 2019-05-31

**Authors:** Paula Bergman, Camilla Munsterhjelm, Anna-Maija Virtala, Olli Peltoniemi, Anna Valros, Mari Heinonen

**Affiliations:** 1 Faculty of Veterinary Medicine, Department of Production Animal Medicine, University of Helsinki, Paroninkuja 20, 04920, Saarentaus, Helsinki, Finland; 2 Department of Production Animal Medicine, Faculty of Veterinary Medicine, Research Centre for Animal Welfare, P.O. Box 57, 00014 University of Helsinki, Saarentaus, Helsinki, Finland; 3 Faculty of Veterinary Medicine, Department of Veterinary Biosciences, University of Helsinki, P.O. Box 66, 00014 University of Helsinki, Saarentaus, Helsinki, Finland

**Keywords:** Sow removal, Management, Housing, Multiple correspondence analysis, Hierarchical cluster analysis, Epidemiology

## Abstract

**Background:**

The main objectives of this observational, cross-sectional study were to characterize piglet producing farms in Finland and to investigate how farm profiles are associated with sow culling and mortality.

The study was conducted on 43 farms during 2014. A questionnaire survey was administered in-person and supplemented with observations in the housing facilities. Annual removal figures and average monthly sow inventories were retrieved from a centralized animal data recording system (National Swine Registry) administered by the Finnish Food Authority. Multiple correspondence analysis and hierarchical clustering were used to explore the complex underlying data-driven patterns.

**Results:**

Sow removal varied markedly between farms with an overall average culling percentage of 38.0% (95% CI 34.1–42.0) and a relatively high average mortality percentage 9.7% (95% CI 7.9–11.5). We identified three farm clusters, which differed both in their typologies and removal patterns. Cluster 1 included farms with features indicative of a semi-intensive or intensive kind of farming, such as larger herd and room sizes, higher stocking density and more sows per caretaker. Most of the cluster 1 farms exceeded the investigated cut-off levels for culling and mortality. Cluster 2 farms were estimated to have the best animal welfare among the sample farms based on a combination of environmental indicators (e.g. amount of bedding, rooting and nesting materials, space allowance, pen cleanliness) and the lowest level of sow mortality as an animal-based indicator. Cluster 3 farms followed a strategy of a rather non-intensified system based on the predominance of smaller herd size, lower stocking density and less sows per caretaker, combined breeding and gestation rooms and rare use of farrowing induction. This cluster showed the lowest culling levels within the sample.

**Conclusions:**

This study captures the diversity among Finnish sow farms and provides a baseline assessment of their practices and facilities. Our results support the notion that farm typologies are associated with sow culling and mortality. In summary, the control of suboptimal sow removal cannot be based on single improvements only, because of other limitations within the individual farm resources.

## Introduction

The relationship between a sow’s reproductive performance and longevity is widely demonstrated in the literature [1–4]. However, sow longevity at herd-level continues to be unsatisfactorily addressed. Herd-level factors can modify sow-level outcomes independently of the sow’s characteristics. In order to understand and control sow removal it is necessary to be familiar with its multidimensional nature, in which deficits in one of the factors can be compensated for by better abilities in the others, and vice versa.

Farms within and between countries differ in their housing, gilt management, genetics, herd health status, feeding practices, human-animal handling and caretaker skills, perceptions, replacement strategies and overall production circumstances [5, 6]. Due to the great number of differences between sow housing systems and management any problems become difficult to determine and control [5, 7]. To date, conditions and management practices within farms have mainly been included as separate effects in studies either on the risk factors for the main causes of premature removal or quantifying the direct risks for a sow to exit the herd [8–11] . Careful classification of farms might contribute more efficiently to understanding mechanisms in suboptimal sow removal than the single predictor approach that has dominated past epidemiological research.

Nevertheless, the multitude of current features of animal husbandry has not been extensively described in the literature. European Livestock Farming Systems (LSF) research has put emphasis on increasing knowledge about the diversity of the farming systems [12]. However, the knowledge gap has remained: Stalder et al. [13] called attention to more appropriate evaluation of current sow longevity taking the complexity of the phenomenon including the impact of farm into account. In line with this, Engblom [14] also concluded in 2008 that there is still a need for further research investigating the effects of management and different housing systems on sow longevity. In general, the lack of sufficient data available on the whole range of farm typologies for agricultural system and livestock environmental impact models, their evaluation, development and application to most closely match the conditions of specific farms remains a major limitation, too [15, 16]. The interest in assessing animal welfare from a multifactorial point of view and farms as a whole has markedly increased during the last two decades [17]. However, the challenges related to the heterogeneity of farms have also been recognized in overall guidelines such as those published by EFSA [18].

The OIE (2004) has raised concern on the welfare implications of modern farming. Society’s awareness of the effects of intensive livestock systems is changing aims of research towards sustainability [19] and animal well-being instead of increased productivity [20, 21]. Sow longevity at herd-level is not only a key trait in improving farm profitability but is also associated with overall animal well-being and socio-cultural acceptance of farming [22–24]. Especially involuntary culling for impaired health and on-farm mortality raise welfare issues [6, 25]. Morbidity and mortality are indicators of insufficient freedom from pain, injury or disease, and thus are useful animal-based tools in assessing welfare [17, 25]. In addition, there is increased awareness of the interactions between animal welfare and farmer wellbeing [26, 27].

The primary objective of this observational study was to assess the multifactorial nature of sow farms and identify groups with similar management practices and housing conditions, i.e. “farm profiles” based on data collected through farm visits using multiple correspondence and cluster analyses. The second objective was to provide baseline estimates for sow culling and mortality derived from a centralized animal data recording system (National Swine Registry) and investigate the relationships between sow removal and farm descriptors.

## Materials and methods

### Selection of farms

Details on the recruitment and selection of the study farms are given in Heinonen et al. [28]. Shortly, the study was originally designed to represent Finnish piglet production. All piglet producing farms operated for primary income were eligible to voluntarily participate. After an extensive publicity campaign 43 farms were visited either after having volunteered on their own initiative (*n* = 12) or been contacted by telephone and being convenience sampled (*n* = 31). The final sample encompassed a diversity of farm types including family-managed and farms run jointly by several farmers. They were scattered across Finland apart from Lapland having no piglet production.

### Farm data collection

All farms were visited between February and October 2014 by the first author. The assessment involved two main parts: a face to face interview and detailed farm observations. Each farm visit lasted for an entire day.

A questionnaire was designed to structure the interview. It was in Finnish and encompassed predominantly closed questions or semi-closed ones investigating e.g. farm specific characteristics, biosecurity, health care and production phase specific management. The questions were piloted for clarity, but no validity or repeatability testing could be done due to the limited number of volunteering participants and strict timing. The farm interview was made with the head of the staff on five and the owner on 38 farms and completed in 2 to 6 hours. After the interview, each production unit (breeding, gestation, farrowing) was monitored according to a detailed checklist.

The combined results constitute the data set of the present work. The full questionnaire and the checklist for production facilities are available upon request from the corresponding author.

### Removal data

Registration of pigs is regulated both by Finnish and EU legislation [29]. Each producer reports the monthly animal inventories and their changes three times a year to the National Swine Registry administered by the Finnish Food Authority. All study farms consented to have their removal data analyzed. For each herd, the annual culling and mortality percentages were calculated as the total number of animals sent to slaughter or died on-farm (euthanasia or unassisted death) divided by the average of monthly sow inventories. The overall removal percentage was defined as the summation of the culling and mortality percentages. Total numbers were collected from January through December 2014 for all except for one farm, for which data only until July were considered representative, because of the farmer giving up farming towards the end of the year.

### Data analysis

Data processing and analysis were carried out using RStudio for R software and its packages FactoMineR and FactoExtra especially dedicated to multivariate exploratory data analysis [30–33].

Variables lacking information, presenting too little or too much variability or not supplying relevant information for the purposes of this study were removed from the generated data set.

#### Multiple correspondence analysis (MCA)

Multiple correspondence analysis (MCA) is the multivariable extension of correspondence analysis (CA). It works effectively for large data matrices comprised of any types of categorical variables (binary, ordinal or nominal) at the same time and can be used as a preliminary study before modelling, as a pre-processing step or as a main objective in statistical analyses [34]. Its core idea is to provide graphical outputs to uncover variable relationships which would otherwise not be detected through pairwise analyses, yielding a simplification of complex data as a small number of perpendicular dimensions, i.e. axes, variables or components [35]. Two-dimensional displays summarize the proximities between the subjects and locate the variable categories at the centers of the group of subjects that belong to that particular category [36]. Subjects with many frequent variable categories are located near the origin of the dimensions. On the contrary, the further a subject lies from the origin, the greater the deviation is from the expected, or the sample average pattern [35, 36].

#### Variable selection

**Fig. 1:**
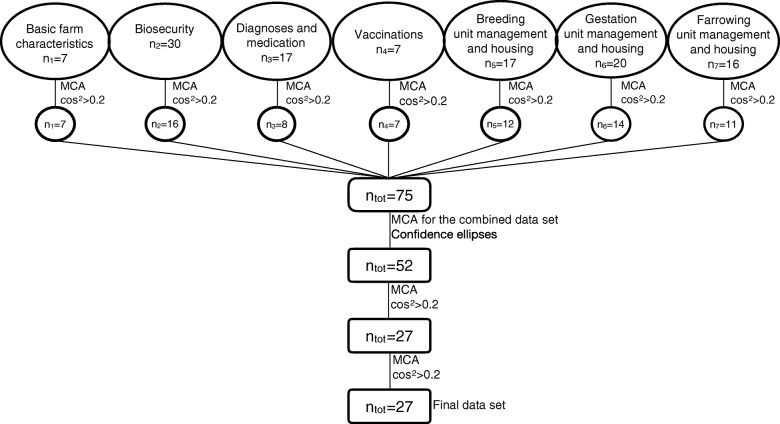
MCA flowchart. Flowchart describing the pre-processing steps for the construction of the final multiple correspondence analysis (MCA) to explore the farm descriptors and removal patterns visually and to be used for further classification purposes. In MCA, cos^2^ can be interpreted as the quality of representations of variable categories. All variables having any category with a cos^2^ > 0.2 in at least one of the first three dimensions remained to be used in further analyses. Small n indicates the number of variables and the subscript the corresponding block

Continuous variables were categorized using the overall median values or what seemed most discriminating or yielded an appropriate number of items in each category. Due to the large number of variables, selection was necessary before performing further analyses. To identify the most representative variables a previously reported stepwise strategy [37] was followed as shown in Fig. 1. In the first step, seven blocks of variables were presented separately to the MCA. In MCA the squared cosine can be interpreted as a measure of the quality of a variable category [32]. Thus, the cos^2^-criterion (cos^2^ > 0.2) was used to select representative variables in each of the pre-processing step. Some variables considered important in discriminating the farms, were maintained even when not meeting the cos^2^-criterion in the first round.

After carrying out the stepwise analyses the remaining variables were merged, MCA was repeated, and confidence ellipses were used to identify the discriminating variable categories through graphical examination [32]. After having merged the overlapping categories using the ellipses, MCA was repeated, and the cos^2^-criterion was applied twice to further focus the analyses. The variables finally selected, i.e. the most informative ones on the basis of their considered contribution in characterizing the farms, are presented in Table 1 with detailed information.

**Table 1:** Variables and variable categories used in the construction of the final multiple correspondence analysis (MCA)

Variable code	Description (“category”)
Type	Type of production including the breeding and farrowing of sows and thereafter either selling the pigs as feeders or raising pigs to their slaughter weight (“far-feed” = farrow-to-feeder, “far-fin” = farrow-to-finish)
Herdsize	Number of sows based on the 25th and 75th sample percentiles (“Herd<103”, “Herd103–635”, “Herd >635”)
Sows/caretaker	Sows per caretaker-ratio as the number of sows divided by the number of caretakers based on the 25th and 75th sample percentiles (“Sows/caretaker <58”, “Sows/caretaker =58–147”, “Sows/caretaker>147”)
BR_size	Number of sows in the same room in the breeding unit (“BR_size<20”, “BR_size20–50”, “BR_size >50”, “BR_sizeall” = all sows in the same room)
GE_size	Number of sows in the same room in the gestation unit based on the 25th and 75th sample percentiles (“GE_size<50”, “GE_size50–151”, “GE_size>151”)
GE_group	Number of sows in the same group in the gestation unit based on the 50th sample percentile (“GE_group<11”, “GE_group>10”)
GE_area	Measured area (m^2^) divided by the number of sows in the gestation unit based on the 50th and 75th sample percentiles (“GE_area < 3”, “GE_area3–3.7”, “GE_area > 3.7”)
FAR_pen	Measured farrowing pen size (m^2^) categorized based on the 75th sample percentiles (“FAR< 5.26”, “FAR> 5.26”)
BR_GE_comb	Housing of the weaned and gestating sows in the same room (“no”, “yes”)
BR_type	Type of the feeding system in the breeding unit (“stall_L” = locked stalls,“trough” = pen with trough feeding)
GE_type	Housing in the gestation unit (“loose” = group housing with electronic transponders, “pen” = pen with trough feeding, “pen_stall” = pen with feeding stalls, “pen_stallL” = pen with lockable feeding stalls)
FAR_ind	Frequency of farrowing induction according to the farmer (“IND_never”, “IND_sometimes”, “IND_always”)
FAR_OX	Frequency of farrowings where oxytocin is used after the onset of parturition out of 10 farrowings according to the farmer (“OX_0–3/10”, “OX_4–7/10”, “OX_ > 7/10”)
FAR_AIAO	All in all out practice in the farrowing unit (“no”, “yes”)
BR_floor	Flooring in the breeding unit (“all solid”, “partly_slatted”)
GE_floor	Flooring in the gestation unit (“ > 20% slatted”, “< 20% slatted”)
FAR_floor	Flooring in the farrowing pen for the sow (“solid”, “partly slatted”, “all slatted”)
BR_m GE_m	Manure management (“slurry”, “dry/mix” = dry or a combination of dry manure management and slurry)
BR_bed GE_bed FAR_bed	Material added to the pens to absorb liquids and scored according to (1) the amount and (2) the dryness of the material (“inadqt” = inadequate, i.e. the bedding material, if at all present, is wet, “some” = mostly dry or almost dry and there is enough to form small piles, “a lot” = mostly dry and there is enough to form large piles)
GE_root FAR_root	Enrichment material given to the sows to facilitate manipulative beviour including both bedding-type materials such as straw, hay, peat or newspaper and/or solid objects, such as chains or rope. (“inadqt” = inadequate, i.e. not enough bedding-type material to form small piles, “some” = not enough bedding-type material or solid objects for all sows to manipulate at the same time, “a lot” = enough bedding-type material to allow all sows the possibility to manipulate the material or the objects simultaneously or bedding material scored as “a lot” and considered suitable also for manipulative behaviour (including materials such as peat, straw or sawdust))
FAR_nest	Frequency and amount of nesting material use in the farrowing pen according to the farmer (“inadqt” = inadequate, “some”, “a lot”)
BR_ GE_ FAR_	Approximation of the proportion of the lying surface that is wet with old faeces (“cleaner” ≤10% dirty, “dirtier” > 10% dirty)

#### Supplementary variables

Supplementary variables do not intervene in the MCA construction. They can be added on the graph to provide visualization of their distribution along the dimensions [35]. In order to see how different levels of culling and mortality relate to the farm descriptors and to identify potential clinically relevant cut-off points, we dichotomized both continuous figures using different threshold values. The final transformations 5% (M_5), median (M_med) and 15% (M_15) for mortality and 30% (C_30), median (C_med) and 50% (C_50) for culling as well as farm type were added to the analysis as supplementary variables.

#### Hierarchical cluster analysis (HCA)

In addition to exploring the data and the removal patterns visually, MCA was also used as a pre-processing step for classification purposes. A hierarchical cluster analysis was carried out on the MCA solution in order to sort subjects into clusters based on the level of similarity within and between members of different clusters aiming at the least within-cluster inertia, i.e. variance, and the greatest between them [34, 35]. The characterization of each cluster was tested by comparing variable distributions (hypergeometric test) to identify which sub-categories were over-represented or under-represented in the sample [34].

## Results

The final data included 22 farrow-to-feeder and 21 farrow-to-finish farms. Altogether, the sample farms housed 18,378 sows, which represented a 16% coverage of the national sow inventory including 115,135 sows in 2014. The lower farm/herd size quartile had less than 103 and the upper one more than 685 sows. These data-driven levels were used to describe the study farms as smaller, intermediate and larger in herd size. Compared with the national average in 2014, larger herd sizes were over-represented in the study sample: the average herd size of the study farms was 263 and the median was 427, whereas the average across all the Finnish farms was 72 and the median was 151 sows.

### Multiple correspondence analysis (MCA)

**Fig. 2:**
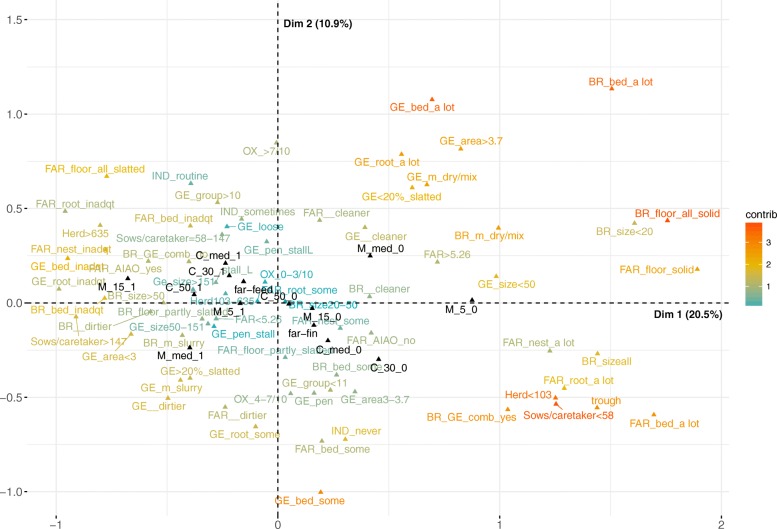
MCA graph of the farm descriptors with different levels of removal. Multiple correspondence analysis (MCA) graph showing the relations between management practices and housing conditions (gradient-coloured) and removal levels (in black) mortality <5% (M_5), <median (M_med) and < 15% (M_15) and culling < 30% (C_30), <median (C_med) and < 50% (C_50) in 43 farms in Finland, 2014. The two perpendicular coordinate axes are referred to as Dim1 (x) and Dim2 (y). To interpret the graph, the dark orange coloured categories are considered to have the strongest contributions, whereas the light blue ones the least, and the points close together in the same quadrants along a similar direction from the centroid would be indicative of possible associations. The acronyms of the farm variable categories are specified in Table 1

The first two dimensions of the final MCA of the farms accounted for 31.4% of the dispersion of the data, i.e. variance or inertia. Figure 2 presents the MCA solution for the variable categories. Only the most informative variables to characterize the farms identified using the stepwise procedure are included (Fig. 1, Table 1).

The first dimension accounted for 20.5% of the variance and was best described by the number of sows per caretaker and herd size. In addition, the variables capturing information about the flooring, the use of bedding material and room size in the breeding unit, the type of flooring, the use of bedding and nesting materials in the farrowing unit, and the use of bedding material in the gestation unit and space allowance described the first dimension.

The most characteristic variables of the second dimension accounting for 10.9% of the variance were mainly related to gestation unit: use of bedding and rooting materials, manure management, group size and flooring. In addition, the use of farrowing induction contributed to the description of this dimension 2.

The categorized removal figures are plotted on the MCA solution as supplementary variables and can be interpreted by their co-location with the farm descriptors (Fig. 2). The variables representing culling percentages below the median or 30% are located in the lower right quadrant, whereas the ones above 30%, the median and 50% are all located in the upper left quadrant. The coordinates of the variables representing the increasing levels of mortality shift progressively from the positive to the negative side of the first dimension.

### Hierarchical cluster analysis (HCA)

**Fig. 3:**
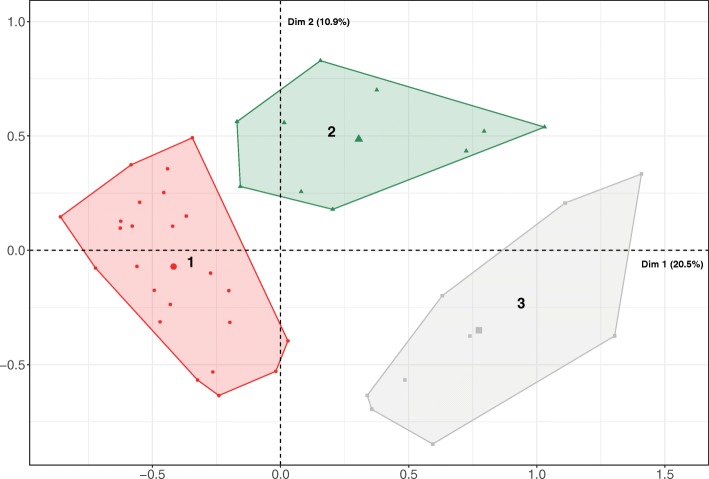
Farm clusters on the MCA graph. Representation of the 3 clusters on the two first dimensions obtained after multiple correspondence and hierarchical cluster analyses of 43 Finnish farms, 2014. The two perpendicular coordinate axes are referred to as Dim1 (x) and Dim2 (y). Cluster 1 is represented in red, cluster 2 in green and cluster 3 in grey

Based on the decrease in within-group inertia, the hierarchical cluster analysis revealed 3 clusters (Fig. 3). Tables 2, 3 and 4 show the distribution of farm descriptors and Table 5 of removal in each cluster together with the total farm sample with notations of significant variation. For example, the cluster 3 farms were characterized by being smaller: there were more smaller farms in this cluster than in the others. Altogether 72.7% of the smaller farms belonged to cluster 3 and 88.9% of the farms in cluster 3 were smaller. These percentages are high considering the overall frequency of smaller farms that was 25.6%. The hypergeometric test was significant (*p* < 0.05) [34].

**Table 2:** Basic farm characteristics among the 3 farm clusters

	Cluster			Total
	1 (*n* = 24)	2 (*n* = 10)	3 (*n* = 9)	*n* = 43
Variable	n (%)	n (%)	n (%)	n (%)
Farm type = farrow to feeder	13 (54.2)	5 (50.0)	4 (44.4)	22 (51.2)
Herd size (number of sows)				
<103	1 (4.2)**	2 (20.0)	8 (88.9)**	11 (25.6)
103–635	13 (54.2)	7 (70.0)	1 (11.1)*	21 (48.8)
>635	10 (41.7)**	1 (10.0)	0 (0.0)*	11 (25.6)
Number of sows per caretaker				
<58	1 (4.2)**	2 (20.0)	9 (100.0)**	12 (27.9)
58–147	12 (50.0)	8 (80.0)*	0 (0.0)**	20 (46.5)
>147	11 (45.8)**	0 (0.0)*	0 (0.0)*	11 (25.6)
Breeding unit room size (number of sows)				
<20	0 (0.0)	1 (10.0)	2 (22.2)	3 (7.0)
20–50	7 (29.2)	3 (30.0)	1 (11.1)	11 (25.6)
>50	17 (70.8)*	5 (50.0)	2 (22.2)*	24 (55.8)
all sows in the same room	0 (0.0)*	1 (10.0)	4 (44.4)**	5 (11.6)
Gestation unit room size (number of sows)				
<50	2 (8.3)*	3 (30.0)	6 (66.7)**	11 (25.6)
50–151	13 (54.2)	6 (60.0)	2 (22.2)	21 (48.8)
>151	9 (37.5)*	1 (10.0)	1 (11.1)	11 (25.6)
Gestation unit group size ≥11 sows	12 (50.0)	7 (70.0)	1 (11.1)*	20 (46.5)
Gestation unit floor space per sow (m^2^)				
<3	18 (75.0)**	0 (0.0)**	2 (22.2)	20 (46.5)
3–3.7	5 (20.8)	3 (30.0)	4 (44.4)	12 (27.9)
>3.7	1 (4.2)**	7 (70.0)**	3 (33.3)	11 (25.6)
Farrowing pen size ≥5.26m^2^	2 (8.3)**	6 (60.0)*	4 (44.4)	12 (27.9)

Categories that are statistically significantly over-represented (bold) or under-represented in the cluster than in the overall frequency are specified as * (*p* < 0.05) and ** (*p* < 0.01). Table 1 presents detailed variable information

**Table 3:** Farm structure, farrowing related medication and farrowing unit all-in-all-out practice among the 3 farm clusters

	Cluster			Total
	1 (*n* = 24)	2 (*n* = 10)	3 (*n* = 9)	*n* = 43
Variable	n (%)	n (%)	n (%)	n (%)
Combined breeding and gestation units = yes	2 (8.3)**	3 (30.0)	7 (77.8)**	12 (27.9)
Breeding unit feeder type				
locked stall	24 (100.0)**	9 (90.0)	3 (33.3)**	36 (83.7)
trough	0 (0.0)**	1 (10.0)	6 (66.7)**	7 (16.3)
Gestation unit pen design				
group housing with electronic transponders	1 (4.2)	1 (10.0)	1 (11.1)	3 (7.0)
pens without stalls	9 (37.5)	1 (10.0)*	6 (66.7)*	16 (37.2)
pen with stalls	2 (8.3)	1 (10.0)	0 (0.0)	3 (7.0)
pen with locked stalls	12 (50.0)	7 (70.0)	2 (22.2)	21 (48.8)
Farrowing induction				
never	8 (33.3)	2 (20.0)	7 (77.8)*	17 (39.5)
sometimes	13 (54.2)	7 (70.0)	2 (22.2)*	22 (51.2)
always	3 (12.5)	1 (10.0)	0 (0.0)	4 (9.3)
Use of oxytocin during farrowing				
0–3/10 farrowings	10 (41.7)	2 (20.0)	5 (55.6)	17 (40.0)
4–7/10 farrowings	10 (41.7)	4 (40.0)	4 (44.4)	18 (41.9)
> 7/10 farrowings	4 (16.7)	4 (40.0)	0 (0.0)	8 (18.6)
Farrowing unit all-in-all-out practice = yes	14 (58.3)*	3 (30.0)	1 (11.1)*	18 (41.9)

Categories that are statistically significantly over-represented (bold) or under-represented in the cluster than in the overall frequency are specified as * (*p* < 0.05) and ** (*p* < 0.01). Table 1 presents detailed variable information

**Table 4:** Flooring, manure management, use of enrichment materials and pen dirtiness among the 3 farm clusters

	Cluster			Total
	1 (*n* = 24)	2 (*n* = 10)	3 (*n* = 9)	*n* = 43
Variable	n (%)	n (%)	n (%)	n (%)
Breeding unit proportion of solid floor = all solid	0 (0.0)**	3 (30.0)	4 (44.4)*	7 (16.3)
Gestation unit proportion of solid floor ≥80%	4 (16.7)**	9 (90.0)**	4 (44.4)	16 (37.2)
Farrowing unit proportion of solid floor				
fully slatted	9 (37.5)*	2 (20.0)	0 (0.0)*	11 (25.6)
partly slatted	15 (62.5)	7 (70.0)	6 (66.7)	28 (65.1)
all solid	0 (0.0)*	1 (10.0)	3 (33.3)*	4 (9.3)
Breeding unit manure management = dry or combination	3 (12.5)**	5 (50.0)	5 (55.6)	13 (30.2)
Gestation unit manure management = slurry	21 (87.5)**	1 (10.0)**	4 (44.4)	26 (60.5)
Breeding unit amount of bedding material				
a lot	0 (0.0)**	4 (40.0)*	2 (22.2)	6 (14.0)
some	8 (33.3)	4 (40.0)	7 (77.8)*	19 (44.2)
inadequate	16 (66.7)**	2 (20.0)	0 (0.0)**	18 (41.9)
Gestation unit amount of bedding material				
a lot	1 (4.2)**	10 (100.0)**	2 (22.2)	13 (30.2)
some	10 (41.7)	0 (0.0)**	7 (77.8)*	17 (40.0)
inadequate	13 (54.2)**	0 (0.0)*	0 (0.0)*	13 (30.2)
Farrowing unit amount of bedding material				
a lot	0 (0.0)*	0 (0.0)	5 (55.6)**	5 (11.6)
some	6 (25.0)	2 (20.0)	3 (33.3)	11 (25.6)
inadequate	18 (75.0)*	8 (80.0)	1 (11.1)**	27 (62.8)
Gestation unit amount of rooting material				
a lot	3 (12.5)**	10 (100.0)**	3 (33.3)	16 (37.2)
some	14 (58.3)	0 (0.0)**	6 (66.7)	20 (46.5)
inadequate	7 (29.2)*	0 (0.0)	0 (0.0)	7 (16.3)
Farrowing unit amount of rooting material				
a lot	1 (4.2)*	0 (0.0)	5 (55.6)**	6 (14.0)
some	18 (75.0)	10 (100.0)*	4 (44.4)*	32 (74.4)
inadequate	5 (20.8)*	0 (0.0)	0 (0.0)	5 (11.6)
Farrowing unit amount of nesting material				
a lot	0 (0.0)*	1 (10.0)	3 (33.3)*	4 (9.3)
some	11 (45.8)	7 (70.0)	6 (66.7)	24 (55.8)
inadequate	13 (54.2)**	2 (20.0)	0 (0.0)*	15 (34.9)
Breeding unit pen dirtiness = dirty	14 (58.3)*	3 (30.0)	1 (11.1)*	18 (41.9)
Gestation unit pen dirtiness = dirty	16 (66.7)**	1 (10.0)*	2 (22.2)	19 (44.2)
Farrowing unit pen dirtiness = dirty	13 (54.2)	2 (20.0)	4 (44.4)	19 (44.2)

Categories that are statistically significantly over-represented (bold) or under-represented in the cluster than in the overall frequency are specified as * (*p* < 0.05) and ** (*p* < 0.01). Table 1 presents detailed variable information

**Table 5:** Sow removal patterns based on different cut-off levels among the 3 farm clusters

	Cluster			Total
	1 (*n* = 24)	2 (*n* = 10)	3 (*n* = 9)	*n* = 43
Variable	n (%)	n (%)	n (%)	n (%)
Culling > 30%	19 (79.2)*	8 (80.0)	2 (33.3)*	29 (67.4)
Culling > median	15 (62.5)*	5 (50.0)	1 (11.1)*	21 (48.8)
Culling > 50%	4 (16.6)	1 (10.0)	0 (0.0)	5 (11.6)
Mortality > 5%	23 (95.8)*	6 (60.0)*	7 (77.7)	36 (83.7)
Mortality > median	16 (66.7)*	2 (20.0)*	4 (44.4)	22 (51.2)
Mortality > 15%	7 (29.2)*	0 (0.0)	1 (11.1)	8 (18.6)

Categories that are statistically significantly over-represented (bold) or under-represented in the cluster than in the overall frequency are specified as * (*p* < 0.05) and ** (*p* < 0.01)

The type of production (farrow-to-feeder and farrow- to-finish producers), which was not a representative variable in MCA construction, was uniformly distributed in all clusters (Table 2). Herds in cluster 1 were most frequently larger, having more than 635 sows and more sows per caretaker, whereas for cluster 3 farms smaller herds and less sows per caretaker were very common. Cluster 2 did not differ in herd size, but an average number of sows per caretaker was most common compared with the other clusters.

Larger room sizes were mostly favored in cluster 1 farms both in the breeding and gestation units, where also higher stocking density was most common compared with the other clusters. Their sows were also most likely to have smaller farrowing pens. On the contrary, for cluster 3 farms it was most common to keep the gestating sows in rooms smaller than 50 animals as well as in smaller groups in comparison with the other clusters. No statistical associations existed with regard to cluster 2, but it was observed that larger breeding and intermediate gestation unit room sizes and larger gestation sow group sizes were common. However, a very common feature of this cluster was its having the most space allowance in the gestation unit, and more than half of these farms also had larger farrowing pens compared with the farms of the other clusters.

The use of a combined breeding and gestation unit was common in cluster 3 farms, whereas it was hardly ever observed in cluster 1 farms (Table 3). Cluster 1 farms always used locked stalls in the breeding unit, whereas trough feeding was most common in cluster 3 farms. Cluster 2 did not differ significantly from the other two clusters with regard to feeding practices, but similarly the locked stalls were mostly used. The four distinct gestation unit types (group housing with electronic transponders, pens without stalls with trough feeding, pens with stalls and locked stalls) were mainly uniformly distributed, apart from pens without stalls, which were mostly common in cluster 3 farms.

The use of farrowing induction and oxytocin during farrowing were quite uniformly distributed in the clusters 1 and 2. Cluster 3 farms hardly ever used induced parturition.

Variables describing the flooring, manure management and the use of bedding, rooting and nesting materials varied between the clusters (Table 4). In cluster 1 farms the floor was never completely solid in either the breeding unit or the farrowing pens. Similarly, in general more than 20% slatted floors in the gestation unit were commonly observed, and manure was mostly managed as slurry in these farms. An inadequate amount of bedding material predominated in the breeding and gestation units as well as farrowing pens compared with the other two clusters. In addition, dirty pens were more likely to be observed in the cluster 1 breeding and gestation units than in the other clusters.

A solid floor in the gestation unit characterized cluster 2 farms and manure was most often managed as dry. Furthermore, these farms were the most frequent users of a lot of bedding material in the breeding and gestation units, where the most frequent use of a lot of rooting material was also observed. Cluster 2 gestation pens were hardly ever evaluated as dirty.

A completely solid floor in the breeding unit was most common in cluster 3 and was also most frequently observed in their farrowing pens. Cluster 3 farms seemed most active in providing at least some bedding, a lot of rooting and a lot of nesting materials in the farrowing pens in comparison with the cluster 1 and 2 farms.

### Removal figures

Significant variation in the exceedance of the different dichotomized culling (30%, median, 50%) and mortality (5%, median, 15%) cut-off levels of sow removal was found over the three clusters (Table 5). Farms from cluster 1 most frequently had their culling levels above 30% and the median, and their mortality levels above all the investigated levels compared with clusters 2 and 3. The overall frequency of on-farm mortality above 15% was 18.6%. Altogether 87.5% of the farms exceeding this level belonged to cluster 1 and 37.5% of the farms in cluster 1 represented this exceedance.

Across the clusters, cluster 2 farms were most likely to have their mortality levels both under the median and under 5%. On-farm mortality was below the 5% cut-off in 16.3% of the farms. Altogether 57.1% of these farms belonged to cluster 2 and 40.0% of the cluster 2 farms remained below the 5% mortality level.

Cluster 3 farms differed from the other farms in having their culling levels most often both under 30% and the median. The overall frequency of culling below the level of 30% was 32.6%. Altogether 50% of these farms belonged to cluster 3 and 66.7% of cluster 3 farms had their culling levels below 30%.

Across the 43 herds, the average overall sow removal percentage was 47.7% (95% CI 43.3–52.2). The average proportion of sows sent to slaughter was 38.0% (95% CI 34.1–42.0) and the median was 35.0%. On average, 9.7% (95% CI 7.9–11.5) of the sows died on-farm and the median mortality was 8.3%. The annual culling and mortality percentages in different herds ranged from 14.0 to 80.0 and from 0.0 to 26.8, respectively.

**Fig. 4:**
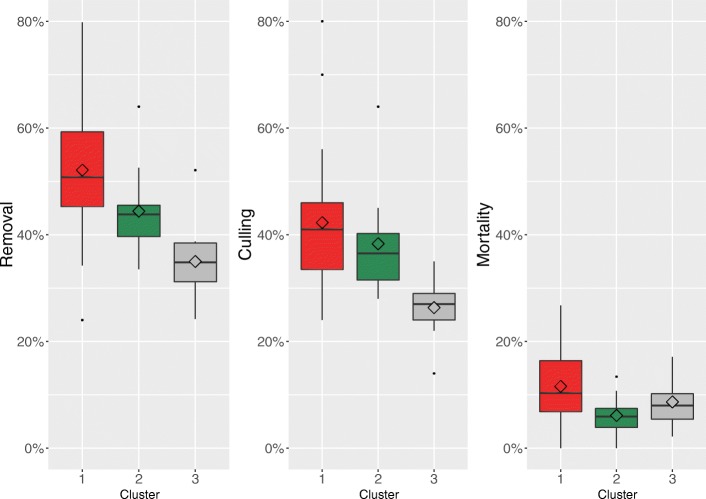
Overall removal, culling and mortality by cluster (means included). Boxplots showing median (central thick lines), 25 and 75% quartile ranges around the median (box width) and mean (diamond) of the three separate removal figures (removal, culling, mortality) for each farm cluster (1 in red, 2 in green and 3 in grey) obtained after multiple correspondence and hierarchical cluster analyses of 43 Finnish farms, 2014

Figure 4 illustrates the removal percentage comparisons for each cluster. In cluster 1 the average culling percentage was 42.3% (95% CI 36.7–47.8) and mortality percentage was 11.6% (95% CI 8.9–14.3). In cluster 2 the average culling and mortality percentages were 38.3% (95% CI 30.8–45.8) and 6.1% (95% CI 3.3–8.9), respectively. An average culling percentage of 26.3% (95% CI 21.5–31.2) was calculated for cluster 3, whereas its average mortality percentage was 8.7% (95% 4.9–12.4). Cluster 3 had a statistically significantly lower culling percentage compared with clusters 1 and 2, whereas cluster 2 had lower mortality compared with cluster 1.

## Discussion

### General remarks

To the best of our knowledge, the present study is the first one showing clusters of piglet producing farms with contrasting patterns of management practices and housing, investigating them with special emphasis on sow removal and demonstrating associations between farm typologies, culling and mortality. Although focused on Finnish production, we expect the approach to be beneficial to other pork producing countries.

### Removal figures

The findings of this study showing great variation among farms are in line with scientific literature, but in interpreting the values the reader must be meticulous as the means of defining, calculating and reporting removal differ between studies [6, 9, 10, 38–40]. For comparison and monitoring purposes a contextualization within and between countries is needed [38].

We quantified the magnitude of sow culling and mortality (animals euthanized or found dead) based on regular, mandatory recordings. These centralized animal register databases should be continuously checked by competent authorities [29]. We also used a more precise approach to average the herd animal inventory at monthly intervals over the year as is suggested to improve the quality and clarity of reporting removal [38]. Thus, we believe that our estimates have medium to high validity and reliability. We show that animal registration data can be utilized for other purposes than those for which they are originally being collected, i.e. for benchmarking removal levels at the herd and national levels and to set future goals. Further research is needed to assess the suitability and availability of these data also for multidimensional animal welfare monitoring purposes [41, 42].

In this study, the percentage for sows leaving the herds remained under 50% in 60% of the farms, whereas approximately one in three farms had the removal percentage under the level of 40%. Our results are in concordance with other studies reporting annual replacement rates verging upon 50% [6, 43, 44]. Nevertheless, the goals for replacement levels under emerging production constrains, environmental sustainability and animal welfare objectives need to be reconsidered [19].

The average and median mortality percentages were relatively high, 9.7% (95% 7.9–11.9) and 8.3%, respectively. Only a minority of the farms fell below the 5% cut-off level and exceedance of the 15% cut-off level was also observed. Current sow mortality in different countries has not been reported widely. Three-month mortality rates between Danish herds ranged from 0 to 8% for gestating and from 0 to 25% for lactating sows in one study [10], whereas another reported an average mortality of 12.7% ranging from 5.2 to 34.4% between herds in Denmark [39]. Annual mortality in a selected sample of Swedish herds was 5.2% for sows being euthanized and 2.1% for the ones found dead [6]. Japanese studies have reported mortality rates of 3.9 and 8.9% [40, 45].

We were able to retrieve removal estimates for only 12 months. In order to improve the monitoring of national trends in culling and on-farm mortality and to identify farms with long-standing elevated figures it would be advisable to collect long-term data on a continuous basis. In addition, farmers should be encouraged to separately record the destination for all sows (slaughter, euthanasia, sudden death) with one or more reasons to identify and control farm specific causes and predisposing factors for suboptimal removal [38].

### Clusters

#### Cluster 1

Cluster 1 included larger farms with larger room sizes, less space allowance and more sows per caretaker compared with the other clusters. Their sows were placed in locked stalls after weaning, all-in-all-out was practiced in the farrowing unit and hardly any materials were provided for the sows, which were housed mainly in dirtier pens. These features are more indicative of a semi-intensive or intensive-kind of farming compared with the other farms. Most of the cluster 1 farms exceeded the investigated cut-off levels for culling and mortality and this cluster showed the highest culling percentages across the clusters. Almost one in three farms in this cluster had the on-farm mortality above 15%.

Some, rather old studies have related increased sow removal with increasing herd size [44, 46]. D’allaire et al. discussed though that the differences likely were not based on the herd size alone but were also influenced by general management and culling criteria [44]. In a Danish study, a herd size exceeding 100 sows had triple the mortality compared with farms with fewer than 50 sows [47]. On the contrary, Japanese results linked an increased mortality risk to smaller herds. The authors speculated that the production systems, herd health and sow care programs in the small herds may not have been adequately developed [40].

Locomotory disorders including claw, leg and feet problems are multifactorial in nature and very common [48]. They occur across all management systems, contribute to economic losses in all stages of production and are reported to be one of the main removal reasons and cause of on-farm deaths and euthanasia [6, 48, 49]. Four previously identified risk factors for locomotor disorders were found to be non-uniformly distributed between the clusters: increasing amount of slatted floor [44, 50, 51], poorer floor hygiene [11], less space per animal and more sows per caretaker [11] were all over-represented in cluster 1 also showing the highest levels of removal. The interpretation of this association should be considered with care, since no data on specific removal reasons were available. However, these findings can serve as a useful reference for future studies investigating housing and management features - separately and synergistically - to ensure locomotory soundness and prevent unplanned removal. [13, 52].

Moreover, a partially slatted floor combined with inadequate quality and hygiene predisposes sows not only to leg and foot injuries and possible subsequent inflammatory processes, but also to bruises and contusions in other parts of the body, which in turn may lead to premature removal [6]. Limited space has been reported to restrict the freedom of movement and may also promote body lesions, especially together with bad flooring conditions [20]. Thus, sows in cluster 1 farms being more at risk of locomotory problems may also be more likely to suffer from traumatic injuries. However, even though the frequency of injuries according to the farmer was recorded in the current study, the variable did not prove to be significant enough to characterize the farms and was thus rejected in the course of MCA graph development.

#### Cluster 2

Cluster 2 farms were mainly intermediate in size and had an average number of sows per caretaker too. Their pregnant group-housed sows most commonly had more space than farms in the other clusters. Furthermore, compared with the other two clusters (1 and 3), the pens were cleaner, and the sows had the possibility of physical separation during feeding due to the frequent use of locked stalls. For culling this cluster represented average farms both when using cut-off levels and percentages. Regarding mortality this cluster differed significantly in all aspects from the other farms. Most of the cluster 2 farms had their mortality levels below the investigated cut-offs. Two in five farms succeeded in keeping mortality below 5% and none exceeded the level of 15%. This cluster also showed the lowest overall mortality percentage compared with the other clusters (Fig. 4).

In comparison with the other clusters, cluster 2 farms used improved facilities especially in the gestation unit, including more floor area and a plentiful use of bedding and rooting materials, both known to be important for sow welfare and common as environmental-based indicators thereof [7, 20, 53, 54]. The housing system is a very important prerequisite of animal welfare, but management is the factor determining the outcome on a given farm. Scientific animal welfare assessment often combines environmental and animal-based information [53, 55]. Mortality is considered an important animal- based welfare indicator [17, 25]. Furthermore, features known to improve the quality of life of animals through enabling them to show natural behaviour with satisfactorily proven herd health status would enhance socio- cultural acceptance [23, 25, 56]. Thus, the present cluster with the lowest mortality can be considered as representing farms with a higher level of animal welfare in comparison with the other clusters.

#### Cluster 3

In comparison with the other clusters, smaller herd, group and room sizes and less sows per caretaker, rare use of all-in-all-out practice in the farrowing unit and very rare use of farrowing induction were all typical characteristics of cluster 3 farms and indicative of a rather non-intensive production. Further, these farms had implemented supplementary welfare-friendly initiatives such as provision of bedding, rooting and nesting materials, which facilitate natural behavioural needs such as foraging, rooting and nest building in each of the sow’s production stage [20, 57]. Most of the cluster 3 farms had their culling levels under 30% and only one exceeded the sample median. This cluster also differed significantly from the other clusters with regard to culling percentage. Furthermore, more than half of the farms in this cluster had their mortality levels under the overall sample median and only one exceeded the 15% cut-off level for on-farm mortality.

Faust et al. demonstrated that systems with the lowest commercial replacement rates are the most profitable [58]. Productivity in a stable pig herd with maintained optimal herd structure and limited number of first parity females is likely to result in a better overall economic status of the herd. Recently, Niemi et al. showed that from an economic point of view a healthy sow should stay in the herd until her 6th through 10th parity [16]. On the other hand, a low sow turnover may lead to economic inefficiency partly due to slow improvement of genetic quality. An in-depth profitability analysis would require the present analysis to be supplemented with herd parity profiles as well as measures of sow performance and farm economic efficiency.

### Limitations

This study relied on interviewee-reported information and secondary data collected for other purposes. In order to decrease the level of potential response bias, additional data were collected by observing the housing facilities. Another source of bias is related to the sampling as the study was volunteer-based. We could only quantify the differences between the volunteers and the population with regard to herd size. Despite these limitations we captured a diversity of farms in terms of management practices, housing conditions and removal patterns, and the methodological approach using MCA and HCA proved useful for profiling the sample farms and associating them with removal patterns. Generalizable causal relationships are not to be inferred from the findings of this observational study.

## Conclusions

Our study highlights important aspects of the complex structure of farms with various plausible interactions and we show that standard recommendations with regard to suboptimal removal applicable to all are not feasible. This study captures the diversity of Finnish sow farms and provides a baseline assessment of their practices and facilities. Our results support the notion that farm typologies are associated with sow culling and mortality. In summary, the control of suboptimal sow removal cannot be based on single improvements only, because of other limitations within the individual farm resources. This study also provides a register-based baseline for sow culling and mortality that can be used to set goals and for monitoring purposes.
